# Relationship between Alcohol Consumption and Eating Disorders in the Tunisian Population

**DOI:** 10.1192/j.eurpsy.2026.10853

**Published:** 2026-07-31

**Authors:** M. Turki, I. Mannoubi, F. Sahnoun, M. A. Megdiche, N. Halouani, S. Ellouze, J. Aloulou

**Affiliations:** Psychiatry B, Hedi chaker Hospital, Sfax, Tunisia

## Abstract

**Introduction:**

Alcohol consumption is a major public health concern and involves multifactorial determinants. Eating disorders (EDs) have been identified as potentially associated with alcohol use.

**Objectives:**

We aimed to explore the relationship between alcohol consumption and EDs.

**Methods:**

This was a descriptive and analytical cross-sectional study including 161 Tunisian participants. It was conducted through an anonymous online questionnaire (Google Forms). Two validated tools were used: the Alcohol Use Disorders Identification Test (AUDIT) to assess alcohol use and the Eating Attitudes Test (EAT-26) to screen for EDs.

**Results:**

The mean age of participants was 30,09 ± 8,45 years, with a sex-ratio (M/F) of 0.41.Among the 63 alcohol consumers, the mean AUDIT score was 6.27 ± 5.7, with 15.9% meeting criteria for alcohol dependence. The mean EAT score was 13.6 ± 13.9, with 20.5% at high risk of EDs. A significant positive correlation was found between AUDIT and EAT scores (r = 0.24; p = 0.002). Participants at high risk for EDs showed higher rates of alcohol dependence (9.1% vs. 0.8%; p = 0.003).

**Conclusions:**

Our findings highlighted the co-occurrence of disordered eating behaviors and alcohol use, underlining the importance of cross-screening and integrated therapeutic approaches.

**Disclosure of Interest:**

None Declared

